# β-Cyclodextrin metal-organic framework as a green carrier to improve the dissolution, bioavailability, and liver protective effect of luteolin

**DOI:** 10.1016/j.ijpx.2024.100250

**Published:** 2024-04-26

**Authors:** Dan Yang, Min Zhao, Yihe Huang, Liwen Chen, Jiqin Fang, Jiaonan Liu, Miao Wang, Chunjie Zhao

**Affiliations:** a School of Pharmacy, Shenyang Pharmaceutical University, Wenhua Road 103, Shenyang, Liaoning Province, China; b Pharmaceutical Department, Liaoning Provincial People's Hospital, Wenyi Road 33, Shenyang, Liaoning Province, China; c School of Public Health, Shenyang Medical College, Shenyang, Liaoning Province, China; d School of Life Science and Biopharmaceutics, Shenyang Pharmaceutical University, Wenhua Road 103, Shenyang, Liaoning Province, China

**Keywords:** Luteolin, Cyclodextrin metal-organic frameworks, Bioavailability, Liver injury

## Abstract

The incidence of acetaminophen-induced liver injury has increased, but effective prevention methods are limited. Although luteolin has hepatoprotective activity, its low solubility and bioavailability limit its applications. Cyclodextrin metal-organic frameworks (CD-MOFs) possess 3D-network structures and large inner cavities, which make them excellent carriers of poorly soluble drugs. In this study, we used CD-MOFs as carriers to improve the dissolution of luteolin and assessed their antioxidant activity, bioavailability, and hepatoprotective effects. Luteolin was loaded into β-CD-MOF, γ-CD-MOF, β-CD, and γ-CD, and characterized by powder X-ray diffractometry (PXRD) and thermogravimetric analysis (TGA). Our results showed that luteolin-β-CD-MOF was the most stable. The main driving forces were hydrogen bonds and van der Waals forces, as determined by molecular simulation. The loading capacity of luteolin-β-CD-MOF was 14.67 wt%. Compared to raw luteolin, luteolin-β-CD-MOF exhibited a 4.50-fold increase in dissolution and increased antioxidant activity *in vitro*. Luteolin-β-CD-MOF increased the bioavailability of luteolin by approximately 4.04- and 11.07-fold in healthy rats and liver injured rats induced by acetaminophen *in vivo*, respectively. As determined by biochemical analysis, luteolin-β-CD-MOF exhibited a better hepatoprotective effect than raw luteolin in rats with acetaminophen-induced liver injury. This study provides a new approach for preventing acetaminophen-mediated liver damage.

## Highlights

• The loading mechanism of luteolin-β-CD-MOF has been elucidated.
• Luteolin-β-CD-MOF achieved a better anti liver injury effect than luteolin.
• β-CD-MOF is a potential excellent drug carrier for oral drug delivery.

## Graphical abstract

**Figure:**
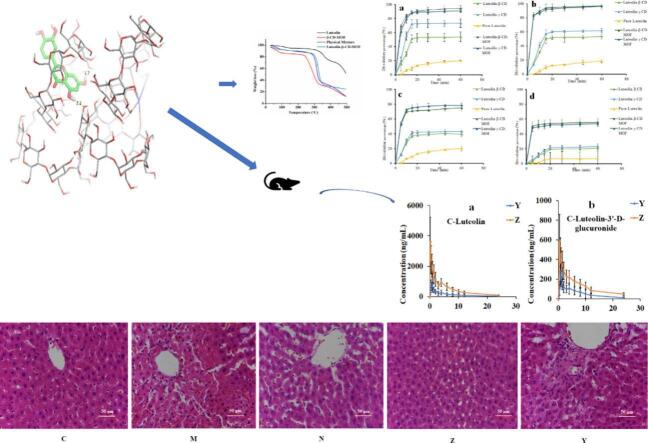

## 1 Introduction

The liver performs many vital functions in the body and is also the primary site for metabolisms of many substances (Tang et al., 2014). Drug-induced liver injury is a threat to human health and may result in serious medical and health problems (Shen et al., 2019). Acetaminophen is a commonly used medicine in clinical practices, but its irrational use is the main cause of acetaminophen-induced liver injury. The excess acetaminophen is metabolized into *N*-acetyl-para-benzoquinone imine *in vivo*, which produces toxic protein adducts, leading to hepatocyte toxicity (Hinson et al., 2010). The incidence and mortality rates of acetaminophen-induced liver injury are increasing (Lancaster et al., 2015). However, methods for treating acetaminophen-induced liver injury are limited. Therefore, researchers have investigated effective strategies to prevent acetaminophen-induced liver injury. Luteolin is a natural flavonoid with hepatoprotective effects (Domitrovic et al., 2009). But its clinical performance is limited by its low water solubility and bioavailability (Carolina and Constanza, 2010). Several strategies, such as co-crystallization (Luo et al., 2019), liposome formation (Li and Cheng, 2016), nano formulations (Wang et al., 2019), and solid dispersion (Zhou et al., 2022), have been implemented to overcome these limitations. However, these strategies have other problems, such as low drug loading, aging, complicated operation techniques, and the need for special instruments. To more effectively prevent liver damage induced by acetaminophen, a simple and eco-friendly method needs to be developed to increase the solubility and bioavailability of luteolin.

Cyclodextrin metal-organic frameworks (CD-MOFs) are edible and renewable materials that have attracted much attention (He et al., 2019a, He et al., 2019b). CD-MOFs not only have the hydrophobic cavities of their parent CDs but also possess additional channels and pores. β-CD-MOF has bowl-shaped pores and “8” type dual channels, which occurs as a left-hand spiral 3D-structure. Therefore, β-CD-MOF has two kinds of voids, namely, the cavity of the original β-CD (6.5 Å in diameter) (Valle, 2004) and the newly formed bowl-like cavity (6 Å in diameter) (Hu et al., 2022). Moreover, γ-CD-MOF has two main types of cavities, which includes pores (17 Å in diameter) formed by (γ-CD)_6_ and pores (8 Å in diameter) formed by the γ-CD pair. These features allow CD-MOFs to easily carry more drug molecules than their parent CDs. Therefore, CD-MOFs have been used as carriers to improve the solubility and bioavailability of drugs (Wei and Chen, 2021; Xu and Wang, 2017). Additionally, γ-CD-MOF is more widely used than β-CD-MOF for encapsulating drugs (Smaldone et al., 2010; Yang and Liu, 2019). He et al., 2019a, He et al., 2019b detected the dual mechanisms of complexation and nano-clusterization for loading azilsartan into γ-CD-MOF. Relevant researches on lansoprazole (Li et al., 2017), myricetin (Chen et al., 2024), and ferulic acid (Xu et al., 2019) have been reported. The mechanisms of γ-CD-MOF encapsulating drugs were almost elucidated in these studies. However, the mechanisms of β-CD-MOF loading drugs are still unclear because the layered stacking structures are unfavorable for loading drugs. Therefore, we studied the mechanism of β-CD-MOF loading luteolin, to explore more applications of β-CD-MOF.

Although many researchers have applied various solubilizing technologies, we focus on new β-CD-MOF as carrier to improve the dissolution rate, antioxidant activity, bioavailability, and liver-protective effects of luteolin. For this purpose, we designed and evaluated luteolin-β-CD-MOF, luteolin-γ-CD-MOF, luteolin-β-CD, and luteolin-γ-CD by conducting several tests *in vitro* and *in vivo*. This is the first study to load luteolin into β-CD-MOF, as well as the first study to elucidate the mechanisms of four carriers loading luteolin simultaneously. This study provides a better way to protect the liver from damage induced by acetaminophen.

## 2 Experimental materials and methods

### 2.1 Materials and reagents

γ-CD was purchased from Hongrun Boshun Technology Co., Ltd. (Beijing, China). β-CD was purchased from Bodi Chemical Reagent Co., Ltd. (Tianjin, China). Potassium hydroxide was purchased from Hengxing Chemical Reagent Co., Ltd. (Tianjin, China). Luteolin, luteolin-3’-D-glucuronide and acetaminophen were purchased from Yuanye Biotechnology Co., Ltd. (Shanghai, China). Polyethylene glycol 20,000 (PEG 20000) was supplied by Macklin Biochemical Technology Co., Ltd. (Shanghai, China). Vitamin C (VC), 2, 2-diphenyl-1-picrylhydrazyl (DPPH) and 2, 2′-azino-bis (3-ethylbenzthiazoline)-6-sulfonic acid (ABTS) were bought from Aladdin Chemical Co. Ltd. (Shanghai, China). Deionized water was prepared in our laboratory.

### 2.2 Preparation of CD-MOFs, luteolin-CD-MOFs, and luteolin-CDs

#### 2.2.1 Preparation of β-CD-MOF and γ-CD-MOF

CD-MOFs were synthesized following a method described in another study (He et al., 2020). Briefly, β-CD (2.84 g) or γ-CD (3.24 g) was mixed with eight equivalents of KOH (1.12 g) in 100 mL of aqueous solution and stirred in a sealed glass reactor, followed by the addition of 60 mL of methanol. This solution was incubated at 60 °C for 6 h to allow crystalline particles to be deposited. Then, 160 mL of 8 mg/mL PEG 20000 methanolic solution was added. This solution was incubated in the refrigerator overnight. The following day, the crystals were harvested by centrifugation at 13,000 rpm for 10 min and washed three times with methanol to remove the residual unreacted reagent. Next, 1% acetic acid-ethanol was added to neutralize the alkaline product. The sediments were immersed in dichloromethane for three days, followed by drying at 50 °C overnight.

#### 2.2.2 Preparation and optimization of luteolin-β-CD-MOF and luteolin-γ-CD-MOF

1.70 g of luteolin was weighted and dissolved in ethanol. And then, a specific amount of β-CD-MOF or γ-CD-MOF was added to the above solution. The mixture was incubated at a set temperature under the constant magnetic stirring. After incubation for a period, the solvent was removed, and the particles were dried at 50 °C overnight. The preparation temperature, reaction time, and host-to-guest ratio were optimized by determining the cumulative dissolution amount in 900 mL of distilled water. More information on the procedure used was presented in Supplementary Materials 1.1 and 1.2.

#### 2.2.3 Preparation of luteolin-β-CD

Firstly, 6.81 g of β-CD was dissolved in 120 mL of distilled water to form a saturated solution at 60 °C. Next, 1.70 g of luteolin was dissolved in 40 mL of ethanol. And then, the above solution was slowly dripped into the β-CD aqueous solution with continuous agitation in a water bath at 60 °C for 6 h. This solution was heated for 0.5 h to remove the organic solvent. Subsequently, the solution was incubated overnight at 4 °C. The precipitated inclusion complex was obtained through filtration, and washed with an appropriate volume of ethanol and water to remove residual substances. The precipitate was dried at 50 °C overnight.

#### 2.2.4 Preparation of luteolin-γ-CD

Approximately 4.64 g of γ-CD was dissolved in 20 mL of distilled water at the room temperature. Then, 1.02 g of luteolin was dissolved in 30 mL of ethanol and added dropwise to the γ-CD aqueous solution with continuous agitation for 6 h. The solution was heated at 50 °C for 0.5 h to remove the organic solvent, followed by incubating overnight at 4 °C. The precipitated inclusion complex was obtained through filtration, and washed with an appropriate volume of ethanol and water to remove the residual substance, respectively. The precipitate was dried at 50 °C overnight.

### 2.3 Characterization

#### 2.3.1 Scanning electron microscopy (SEM)

Samples were fixed on aluminum stubs with conductive double-sided-adhesive tape and then covered with gold. The surface morphology of samples was observed using a scanning electron microscopy (SU8010, Hitachi, Japan) at 5 kV.

#### 2.3.2 Powder X-ray diffractometry (PXRD)

PXRD patterns were recorded by an X-ray diffractometer (Rigaku Ultima IV, Japan). Samples were irradiated with monochromatized CuKa radiation (λ = 1.54 Å). In the angle range of 3–45°, 40 kV tube voltage and 40 mA tube current were selected at a scan speed of 0.1 s/step.

#### 2.3.3 Thermogravimetric analysis (TGA)

Samples were placed in aluminum crucibles, heating from 30 °C to 500 °C at 10 °C/min under a nitrogen flow rate of 30 mL/min. The thermogravimetric information was recorded using a thermal analysis system (NETZSCH, USA).

### 2.4 Determination of loading capacity

All samples were prepared on their own best drug-loading conditions, as determined in 2.2.2. The loading capacities of luteolin-CD-MOFs and luteolin-CDs were determined spectrophotometrically (Ruili Beijing Analytic Instrument Co. Ltd., China). The precisely weighted samples were dissolved in a solvent composed of ethanol and water (1: 1, *v*/v), the absorptions were measured at 350 nm. The loading capacity was the weight percentage of the drug in the drug-loaded samples, and was calculated by the formula (1):

(1) $$\text{Loading capacity}\%=\text{Luteolin determined in sample}/\text{Total weight of}\;{\text{sample}}^{*}100\%$$

### 2.5 Dissolution profile

The dissolution experiments were performed using a RC12AD dissolution system (Tiandatianfa Technology Co., Ltd., China). Samples were carefully weighted, ensuring that they contained approximately equal amounts (10 mg) of the active pharmaceutical ingredient. All experiments were performed under constant stirring at 100 rpm, and the temperature was maintained at 37 °C. The dissolution experiments were performed in 900 mL of distilled water, HCl (pH 1.2), acetate buffer (pH 4.5), and phosphate buffer (pH 6.8), respectively. At predefined time intervals (0, 5, 10, 15, 20, 30, 45, and 60 min), 2 mL of solution was collected from the dissolution medium and analyzed spectrophotometrically (Ruili Beijing Analytic Instrument Co. Ltd. China). An equivalent volume of fresh dissolution medium kept at 37 °C was added, and the cumulative dissolution percentages of the samples were analyzed.

### 2.6 Molecular mechanism

The molecule models of ligand-CD-MOF or ligand-CD were constructed by Discovery Studio 2018 and Amber 18. The Visualizer module in Discovery Studio 2018 (MS, Accelrys Inc., USA) was employed to perform the model construction, and Amber 18 was applied to perform the energy minimization. Amber Tools 19 was used to prepare the starting structures. Binding free energy was calculated by MMPBSA.py scripts embedded in Amber Tools 19. The system topology files were divided by chains and provided to MMPBSA.py script for binding free energy data.

### 2.7 Antioxidant activity *in vitro*

Luteolin has the radical scavenging activity, enhancing its dissolution can improve antioxidant activities (Wang et al., 2019). Therefore, the radical scavenging activities of luteolin-β-CD-MOF and luteolin-γ-CD-MOF were investigated. The details were showed in Table S1. DPPH, ABTS, superoxide anions (O_2_^-•^) scavenging activities were calculated as the formula (2).

(2) $$\text{Scavenging activity}\;\left(\%\right)={\left[1-\left(A_{\mathrm{sample}}-A_{\mathrm{control}}\right)/A_{\mathrm{blank}}\right]}^{*}\;100\%$$

Where A _*sample*_ was the absorbance of sample, A _*blank*_ was the absorbance of blank, and A _*control*_ was the absorbance of the control sample. VC was used as a positive control.

#### 2.7.1 DPPH radical scavenging activity

The DPPH radical scavenging activities of luteolin, luteolin-β-CD-MOF and luteolin-γ-CD-MOF were measured following the method reported by Wu et al. (2015), with slight modifications. Briefly, 2 mL of ethanolic DPPH solution (0.1 mM) was evenly mixed with 2 mL of different concentrations of sample aqueous solutions. The mixed solutions were kept in the dark at the room temperature for 30 min. Finally, the absorbance was measured at 517 nm.

#### 2.7.2 ABTS scavenging activity

Firstly, 38 mg of ABTS was dissolved in 10 mL of distilled water and transferred to a 50 mL flask. Then, 7.5 mg of potassium persulfate was dissolved in 10 mL of distilled water. Equal volumes of both solutions were mixed, and the mixture was incubated in the dark for 12 h. After incubation, the solution was diluted 50 times with methanol to form the ABTS cationic working solution. Next, 0.1 mL of samples at different concentrations was mixed with 4 mL of ABTS cationic working solution and incubated at 37 °C for 2 h in the dark. After incubation, the absorbance was measured at 734 nm.

#### 2.7.3 O_2_^−•^ scavenging activity

The O_2_^–•^ scavenging activity was determined by the pyrogallol auto-oxidation method described in a study by Cheng et al. (2019) with slight modifications. Briefly, 4 mL of Tris-HCL buffer solution (0.05 mol/L) was incubated at 25 °C for 20 min. Subsequently, the sample solution was mixed with the pyrogallol solution, and incubated for 5 min. And then, HCl solution (0.01 mol/L) was added to stop the reaction. The absorbance was measured at 320 nm.

### 2.8 Cytotoxicity assay

Cytotoxicity of β-CD-MOF and γ-CD-MOF was measured on L02 cell using the thiazolyl blue (MTT) method. The cell culture was maintained in RPMI-1640 medium, supplemented with 10% foveal bovine serum and antibiotics (penicillin 100 U/mL, streptomycin 0.1 mg/mL). The culture bottles were maintained in a 37 °C incubator with 5% CO_2_. Then cells were plated on a 96-well flat-bottom microplate at a density of about 10^5^ cells/mL. After incubating overnight, 100 μL aliquots of β-CD-MOF and γ-CD-MOF solution (ranging from 62.5 to 2000 μg/mL) were added into the medium and incubated for 24 h. Then, 10 μL MTT solution (5000 μg/mL) was added and incubated for 4 h. Subsequently, the MTT solution was discarded, 150 μL dimethyl sulfoxide was added to dissolve the formazan crystals. After 10 min, the absorbance was measured at 490 nm using a microplate reader (Thermo Fisher Scientific, USA). Six replicate wells were used for each control and test concentration. No-treated cells were used as control, the cell viability (%) was calculated as the formula (3).

(3) $$\text{Cell viability}\;\left(\%\right)=\left({\mathrm{OD}}_{\mathrm{sample}}-{\mathrm{OD}}_{\mathrm{blank}}\right)/{\left({\mathrm{OD}}_{\mathrm{control}}-{\mathrm{OD}}_{\mathrm{blank}}\right)}^{*}100\%$$

Where OD _*sample*_ was the absorbance of sample, OD _*blank*_ was the absorbance of blank, and OD _*control*_ was the absorbance of the control sample.

### 2.9 *In vivo* pharmacokinetic studies of luteolin (Y) and luteolin-β-CD-MOF (Z) in healthy rats and rats with liver injury induced by acetaminophen

#### 2.9.1 Treatment of animals and UPLC-MS/MS analysis

Male Sprague–Dawley rats (200 ± 20 g) were purchased from Shenyang Pharmaceutical University Lab Animal Research Center. All animal experiments were carried out with the permission of the Institutional Animal Care and Use Committee of Shenyang Pharmaceutical University. To conduct pharmacokinetic studies on healthy rats, 12 rats were randomly divided into two groups (*n* = 6 rats per group). The rats were orally administered luteolin (Y) or luteolin-β-CD-MOF (Z), respectively. The rats were starved for 12 h with free access to water before the experiments. The blood collection time was set at 0, 0.167, 0.5, 0.75, 1, 1.5, 2, 3, 4, 6, 8, 10, 12, and 24 h after administered to healthy rats. To conduct pharmacokinetic studies on rats with liver injury (n = 6 rats per group), the rats were co-administered a single dose of 1000 mg/kg acetaminophen i.p. and orally administered a luteolin (Y) or luteolin-β-CD-MOF (Z), respectively. Blood was collected from the rats with liver injury at 0, 0.167, 0.5, 1, 2, 3, 4, 6, 8, 10, and 12 h after luteolin administration. The single dose of luteolin and luteolin-β-CD-MOF (equal to the active pharmaceutical ingredient) was 100 mg/kg. The blood samples were centrifuged at 13,000 rpm for 10 min to obtain the plasma. The concentrations of luteolin and luteolin-3’-D-glucuronide in plasma were determined by UPLC-MS/MS on a Kromat Universal XB C_18_ column (150 × 2.1 mm, 3.0 μm) at 30 °C. The mobile phase was composed of acetonitrile and 3 mM ammonium acetate aqueous solution (70: 30, *v*/v) with a flow rate of 0.2 mL/min. The injection volume was 2 μL. The multiple reactions monitoring analysis was operated in negative ionization mode to determine the content of luteolin (*m*/*z* 285.7 → 132.7), luteolin-3’-D-glucuronide (m/z 460.6 → 285), and internal standard (quercetin, m/z 301.1 → 254.8).

#### 2.9.2 Liver histological analysis

The liver tissues were fixed in 4% polyformaldehyde for 24 h and stained with hematoxylin-eosin (HE). The morphological observation of liver tissue was assessed under a biological microscope.

### 2.10 *In vivo* liver protective effect of luteolin and luteolin-β-CD-MOF in rats with liver injury induced by acetaminophen

#### 2.10.1 Treatment of animals

30 male rats weighing 200 ± 20 g were placed in rooms with a relative humidity of 40–70% and a relative temperature of approximately 25 °C. The rats were randomly divided into five groups (*n* = 6 rats per group): (1) the control group (C); (2) the liver injured group induced by acetaminophen (1000 mg/kg, i.p.) (M); (3) luteolin (100 mg/kg/d for 10 days) + acetaminophen (1000 mg/kg, i.p.) (Y); (4) luteolin-β-CD-MOF (equal to the active pharmaceutical ingredient 100 mg/kg/d for 10 days) + acetaminophen (1000 mg/kg, i.p.) (Z); and (5) *N*-acetylcysteine (100 mg/kg/d for 10 days) + acetaminophen (1000 mg/kg, i.p.) (N). The Y, Z, and N groups were orally pre-administered luteolin, luteolin-β-CD-MOF, or *N*-acetylcysteine for 10 consecutive days, respectively. One hour after the final administration, the rats in the Y, Z, and N groups were injected intraperitoneally (i.p.) with acetaminophen (1000 mg/kg in sterile saline). The rats in M group were also administered a single dose of acetaminophen (1000 mg/kg, i.p.). All rats were sacrificed 24 h after acetaminophen administration, their blood and livers were collected and stored at −80 °C for subsequent analysis.

#### 2.10.2 Liver histological analysis

The method is same as which described in **2.9.2**.

#### 2.10.3 Biochemical analysis

Serum ALT and AST activities were detected using the kits purchased from JianCheng Bioengineering Institute (Nanjing, China) following their instructions.

### 2.11 Statistical analysis

Data was presented as mean ± SD. Statistical analysis was performed with SPSS 21.0. The pharmacokinetic parameters were calculated by the non-compartmental analysis using the DAS 2.0 software. The significant difference (p) < 0.05 was statistically significant.

## 3 Results and discussions

### 3.1 Optimizing the preparation conditions for luteolin-β-CD-MOF

In a certain range, temperature affects molecular thermal motion and is a key factor in the drug-loading process (Yang et al., 2015). At the same mass ratio and reaction time, the dissolution profile of luteolin-β-CD-MOF prepared at 40 °C was greater than that at 30 °C but similar to that at 50 °C (Fig. S1a). The maximum cumulative dissolution amount was achieved at the prepared temperature of 40 °C. At the same mass ratio and preparation temperature, the dissolution profile of luteolin-β-CD-MOF prepared for 10 h was higher than that for 2 h (Fig. S1b). No further increase in the dissolution profile was recorded when the reaction time increased beyond 10 h. This suggested that loading equilibrium could be reached at a reaction time of 10 h. Additionally, when the mass ratio reached 1:6 (luteolin: β-CD-MOF, *w*/w), the dissolution profile reached its highest value (Fig. S1c). Thus, luteolin-β-CD-MOF was prepared at a mass ratio of 1:6 (luteolin: β-CD-MOF, w/w) under gentle stirring for 10 h at 40 °C in ethanol.

### 3.2 Optimizing the preparation conditions for luteolin-γ-CD-MOF

The dissolution profile of luteolin-γ-CD-MOF prepared at 40 °C was greater than that of at 30 °C and 50 °C (Fig. S1d-f), indicating that the loading process was saturated at 40 °C with the constant mass ratio (Yang and Liu, 2019). The mass ratio (luteolin: γ-CD-MOF, *w*/w) influenced the dynamic balance inside and outside γ-CD-MOF. When the mass ratio was 1:8 (luteolin: γ-CD-MOF, w/w), the dissolution profile remained unchanged, which indicated that the amount of luteolin loaded in γ-CD-MOF was saturated. The influence of reaction time on the loading was investigated in the range of 2–84 h. These results showed that the reaction time affected the amount of luteolin bound to γ-CD-MOF. The dissolution profile suddenly increased from 2 h to 72 h and then reached equilibrium at 72 h. When the mass ratio was 1:8 (luteolin: γ-CD-MOF, *w*/w), the optimal luteolin loading process was achieved through the gentle stirring for 72 h at 40 °C.

The results showed that the preparation time for luteolin-γ-CD-MOF was longer than that for luteolin-β-CD-MOF. This difference probably occurred because the cavities of γ-CD-MOF were larger than those of β-CD-MOF. Therefore, the preparation for luteolin-γ-CD-MOF required more time. The optimal mass ratio for luteolin-β-CD-MOF was 1:6 (luteolin: β-CD-MOF, w/w), while that for luteolin-γ-CD-MOF was 1:8 (luteolin: γ-CD-MOF, w/w). Briefly, β-CD was cheaper than γ-CD, the synthesis cost of luteolin-β-CD-MOF was lower than that of luteolin-γ-CD-MOF. And the prepared procedure for luteolin-β-CD-MOF was more time-saving. Thus, β-CD-MOF was more suitable for luteolin.

### 3.3 Characterization

#### 3.3.1 Characterization of β-CD-MOF and γ-CD-MOF

**Fig. 1:**
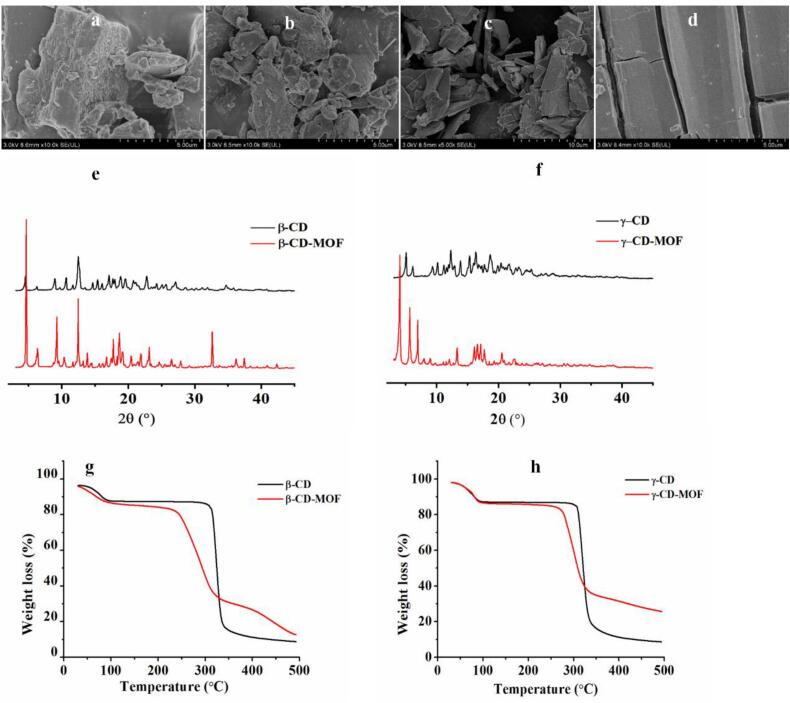
The SEM images of β-CD (a), γ-CD (b), β-CD-MOF (c) and γ-CD-MOF (d). The PXRD patterns of β-CD-MOF (e) and γ-CD-MOF (f). The TGA curves of β-CD-MOF (g) and γ-CD-MOF (h).

SEM images of CD-MOFs and CDs were provided in Fig. 1a-d. We found that the morphological characteristics of the CD-MOFs were different from those of their parent CDs. CDs had irregular shapes and were arranged randomly. While the CD-MOFs had more regular appearances and uneven surfaces. These findings matched those of Ishiwata et al. (2013) and Volkova et al. (2020). The PXRD patterns of the CD-MOFs were shown in Fig. 1e-f. β-CD-MOF had prominent peaks at 4.64°, 6.34°, 8.94°, 9.24°, 10.34°, 12.88° and 18.66°. The main characteristic peaks were located at diffraction angles of 4.04°, 5.66°, 6.92°, 13.32°, 16.60° and 17.10° for γ-CD-MOF. These peaks suggested that β-CD-MOF and γ-CD-MOF were highly crystalline, which was consistent with the findings in other studies (Hu et al., 2021; Jiang and Liu, 2021; Zhao and Guo, 2020; Hu and Wang, 2019). The TGA curves of the CD-MOFs differed from those of their parent CDs (Fig. 1 g-h), which was consistent with the findings in other studies (Qiu et al., 2018; Forgan et al., 2012; Hamedi et al., 2021a, Hamedi et al., 2021b). Three thermal degradation stages were detected in all TGA curves. The onset thermal degradation temperatures of the CD-MOFs in the second degradation stage (approximately 300 °C) were lower than those of their CDs, which indicated that the crystal structures of the CD-MOFs were destroyed before 300 °C (Hu et al., 2021). Due to the presence of metal ions, the weight losses of the CD-MOFs were lower than those of their CDs, which indicated that the thermal stabilities of CD-MOFs were greater than those of the single CDs. These results indicated that β-CD-MOF and γ-CD-MOF were synthesized successfully.

#### 3.3.2 Characterization of luteolin-β-CD and luteolin-γ-CD

**Fig. 2:**
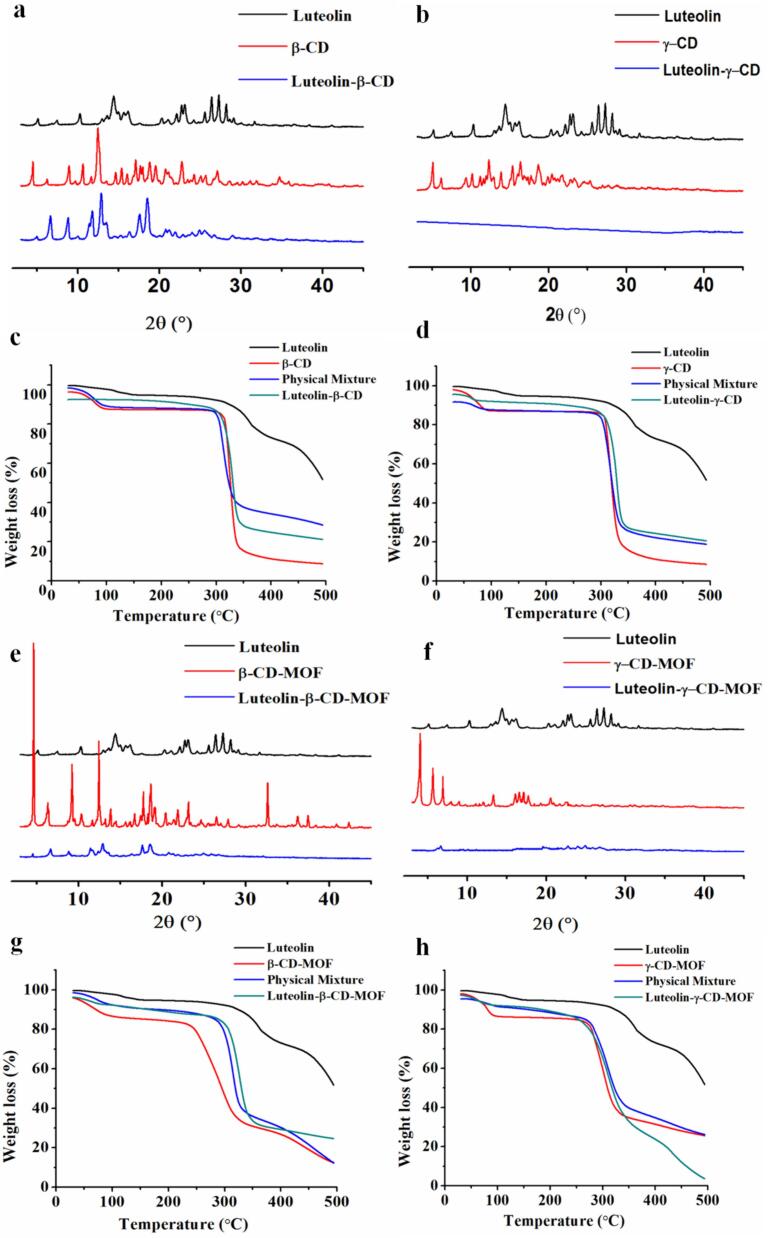
The PXRD patterns of luteolin-β-CD (a) and luteolin-γ-CD (b). The TGA curves of luteolin-β-CD (c) and luteolin-γ-CD (d). The PXRD patterns of luteolin-β-CD-MOF (e) and luteolin-γ-CD-MOF (f). The TGA curves of luteolin-β-CD-MOF (g) and luteolin-γ-CD-MOF (h).

Pure luteolin exhibited sharp and strong reflections at 10.30°, 12.98°, 14.42°, 23.12°, 26.40°, 27.30°, and 28.20°, which indicated that luteolin had high crystallinity. We detected strong peaks of β-CD at 8.90°, 10.66°, 12.48°, 15.38°, 17.10°, 17.96°, 20.76°, 27.10° and weak peaks at 6.20°, 14.72°, 15.50°, 21.22°, and 25.68° (Fig. 2a). These results indicated that β-CD had a typical cage structure, which was consistent with the findings of another study (Liu and Guo, 2017). Some new diffraction peaks were detected for luteolin-β-CD, which indicated that the molecular structure of β-CD was transitioned between the cage and layer (Guo et al., 2011). In the case of γ-CD, strong absorptions were detected at 5.08°, 9.36°, 10.16°, 12.28°, 15.34°, 16.34°, and 18.66° (Fig. 2b). The morphology of luteolin has changed from the crystalline state to the amorphous sate for luteolin-γ-CD. The intrinsic peaks of luteolin were disappeared in luteolin-β-CD and luteolin-γ-CD, which indicated that luteolin was incorporated into the cavities of β-CD and γ-CD. The TGA results showed that the weight losses of luteolin-CDs were lower than those of CDs at all three decomposition stages (Fig. 2c-d). This suggested that a stable association was formed between luteolin and CDs.

#### 3.3.3 Characterization of luteolin-β-CD-MOF and luteolin-γ-CD-MOF

The significant peaks of luteolin were disappeared, and the characteristic peaks of CD-MOFs were detected along with the tiny absorptions in luteolin-CD-MOFs (Fig. 2e-f), indicated that luteolin was incorporated into CD-MOFs successfully. And the crystalline states of CD-MOFs were also changed after the loading process, which was consistent with the findings of another study (Lv et al., 2017). The TGA curves of luteolin-CD-MOFs were different from that of pure luteolin (Fig. 2 g-h), indicated that CD-MOFs changed the thermal behavior of luteolin, and luteolin was loaded into CD-MOFs. The weight losses of luteolin-CD-MOFs were less than those of their free CD-MOFs at the first degradation stage, which occurred before 100 °C. This difference probably occurred because of the presence of luteolin in CD-MOFs (Yang and Liu, 2019). Compared to its physical mixture, the onset temperature of luteolin-β-CD-MOF at the second thermal decomposition stage was shifted to a higher one, and the weight loss of luteolin-β-CD-MOF was reduced. Therefore, compared to its physical mixture, luteolin-β-CD-MOF had a greater thermal stability. These results implied the structure of β-CD-MOF was transferred to a more thermal stable form during the loading process. Additionally, the weight loss of luteolin-γ-CD-MOF was more than that of its physical mixture. Therefore, the crystalline structure of β-CD-MOF had greater thermal stability than that of γ-CD-MOF, which matched the results of another study (Hu et al., 2021). Overall, luteolin-β-CD-MOF had the highest thermal stability among the four complexes examined in this study.

### 3.4 Determination of loading capacity

The loading capacities (wt%) of 4 samples were summarized in Table 1. The loading capacities of luteolin-CD-MOFs were greater than those of luteolin-CDs. This difference probably occurred because the cavities of CD-MOFs were larger than those of CDs, which enabled more drug molecules to be incorporated into their cavities. These results indicated that more interaction bonds were formed between the CD-MOFs and luteolin than between the CDs and luteolin. Additionally, the loading capacity of luteolin-β-CD-MOF (14.67%) was slightly greater than that of luteolin-γ-CD-MOF (11.68%), which indicated that β-CD-MOF had a greater affinity for luteolin than the other three carriers, probably because the features of β-CD-MOF were more suitable for encapsulating luteolin.

**Table 1:** The loading capacity (wt%) of luteolin in samples (Data was presented as mean ± SD, *n* = 6).

	Luteolin-β-CD	Luteolin-γ-CD	Luteolin-β-CD-MOF	Luteolin-γ-CD-MOF
Loading Capacity (wt%)	4.16 ± 0.16	4.97 ± 0.23	14.67 ± 1.38	11.68 ± 2.27

### 3.5 Dissolution profile

**Fig. 3:**
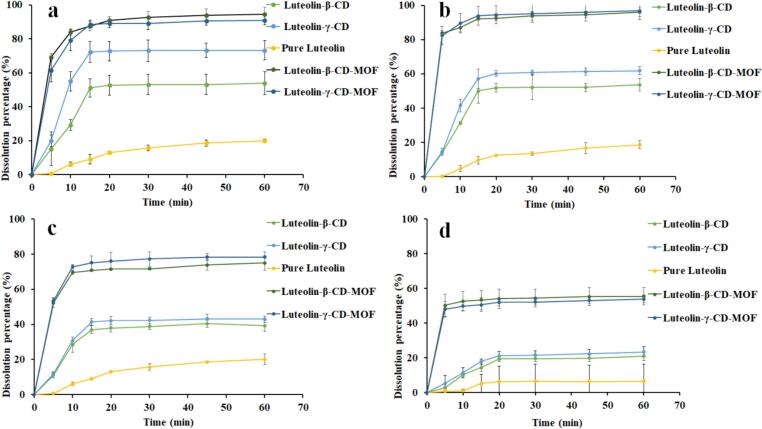
The dissolution profiles of samples in 900 mL of distilled water (a), phosphate buffer (pH 6.8) (b), acetate buffer (pH 4.5) (c), HCl (pH 1.2) (d) (Data was presented as mean ± SD, *n* = 3).

The releasing profiles of samples in four different dissolution media were shown in Fig. 3. Only a small percentage of pure luteolin (20%) was dissolved within 60 min in all tested dissolution media. Compared to raw luteolin, the dissolution profiles of luteolin-CD-MOFs and luteolin-CDs were considerably higher in 4 different dissolution media. For example, we found a 4.50-fold increase in the dissolution amount of luteolin for luteolin-β-CD-MOF and luteolin-γ-CD-MOF in water, whereas the dissolution amount of luteolin increased by 2.72- and 3.71-fold for luteolin-β-CD and luteolin-γ-CD, respectively, compared to raw luteolin. These results indicated that both β-CD, γ-CD, β-CD-MOF, and γ-CD-MOF can improve the dissolution profile of luteolin.

The dissolution profile of luteolin was improved by CDs strategy. Because the hydrophobic inner cavities of β-CD and γ-CD can hold luteolin, which is a hydrophobic molecule. As one can see, the releasing profile of luteolin-γ-CD was slightly greater than that of luteolin-β-CD in water, larger cavity size and greater solubility in water of γ-CD than β-CD could be attributed to this phenomenon.

The dissolution behaviors of luteolin-β-CD-MOF and luteolin-γ-CD-MOF were generally similar, and they both displayed a more enhancement in the dissolution amount than luteolin-CDs. These results indicated that the effectiveness of CD-MOFs was greater than that of CDs in increasing the dissolution of luteolin for several reasons. Firstly, when the complex was in contact with the dissolution medium, the 3D-frameworks of CD-MOFs were dissociated to release luteolin, which promoted the dissolution of luteolin. Secondly, luteolin-CD-MOFs were impelled to form luteolin-CDs, when they were collapsed in the dissolution medium. Besides, when the networks of CD-MOFs were cracked, potassium cations in CD-MOFs increased the drug solubility *via* the salting out effect. Hamedi et al., 2021a, Hamedi et al., 2021b claimed that cations in CD-MOFs help to regulate the releasing behavior of drugs. Most importantly, CD-MOFs have unique structural advantages over CDs, such as larger cavities (Xu and Wang, 2017), structural adjustment (Xiong et al., 2019), and surface adsorption (Iliya and Tatyana, 2020). CD-MOF particles have more exposed sites that can combine with drug molecules. The framework of CD-MOFs also facilitated the formation of drug clusters (Volkova et al., 2020), which were stabilized by CDs during dissolution (He et al., 2019a, He et al., 2019b). The laminated appearance of β-CD-MOFs favored less control over drug release (Liu and Guo, 2017); the more and larger cavities of CD-MOFs facilitated the drug-releasing, which resulted in the above-mentioned phenomenon. Overall, CD-MOFs have multiple mechanisms to improve the dissolution profile of luteolin.

### 3.6 Molecular mechanism

**Fig. 4:**
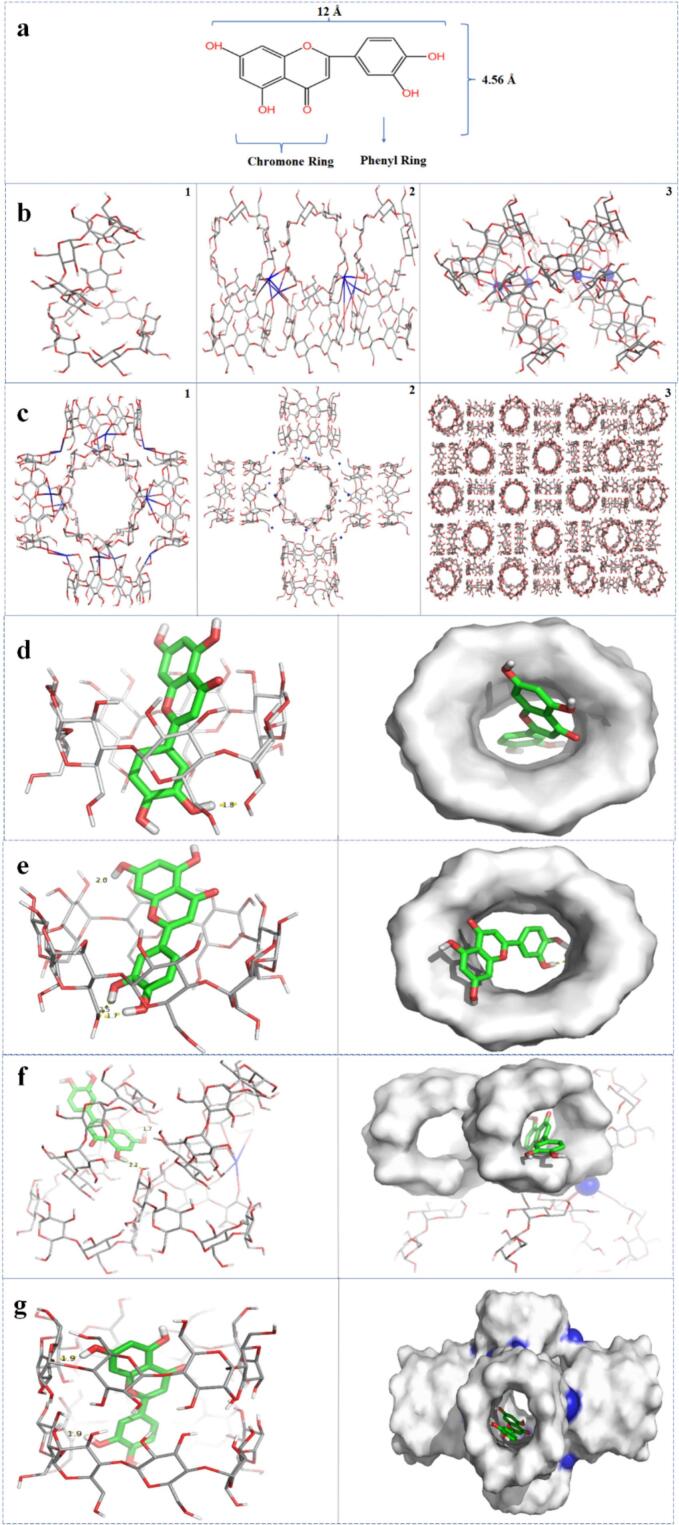
The molecular structure of luteolin (a), 3D-structures of β-CD-MOF (b1–3) and γ-CD-MOF (c1–3). The docking configuration of luteolin-β-CD (d1–2), luteolin-γ-CD (e1–2), luteolin-β-CD-MOF (f1–2) and luteolin-γ-CD-MOF (g1–2).

Luteolin contains a chromone ring and a phenyl ring (Fig. 4a). The size of a luteolin molecule is approximately 12 × 4.56 Å, and luteolin can pass through the pore windows of CDs and CD-MOFs. The structure of mono-β-CD-MOF was formed by two β-CD molecules with bowl-shaped pores (6 Å) (Yang and Liu, 2019), and coordinate bonds were formed (Smaldone et al., 2010; Ke and Zhang, 2019; Furukawa et al., 2012) (Fig. 4b1–3). β-CD-MOF displayed a special configuration with a figure “8” structure (Hu et al., 2021), resulting in steric complementarity between luteolin and β-CD-MOF. Thus, luteolin-β-CD-MOF might have stronger interactions than luteolin-β-CD. There are also K—O coordination bonds in γ-CD-MOF (Fig. 3c1–3). The structure of γ-CD-MOF has a body-centered cubic network of large spherical pores formed by repeating (γ-CD)_6_ cubes, which are connected by numerous channels with about 54% pore vacancies (Forgan et al., 2012). These cavities provide new binding sites for well-suited encapsulating drugs. The phenyl ring of luteolin entered the larger side of β-CD (Fig. 3d1–2), and a hydrogen bond with a bond length of 1.8 Å was formed between the -OH of luteolin and the oxygen atom of β-CD, which helped maintain the structural stability. Similarly, the luteolin molecule entered the cavity from the larger side of γ-CD. Three hydrogen bonds were formed between the -OH of luteolin and the glycosidic oxygen atom of γ-CD, with bond lengths of 2.0 Å, 2.5 Å, and 1.7 Å (Fig. 3e1–2). The number of hydrogen bonds in luteolin-γ-CD system was more than that in luteolin-β-CD system. Hydrogen bonds with bond lengths of 1.7 Å and 2.2 Å were formed between luteolin and β-CD-MOF to support a stable system (Fig. 3f1–2). Two hydrogen bonds were detected for luteolin-γ-CD-MOF, and both the bond distance was 1.9 Å (Fig. 3g1–2). The luteolin molecule was loaded into the hydrophobic cavities of γ-CD pairs (8 Å in diameter), rather than the cages of γ-CD-MOF (17 Å in diameter).

The phenyl ring of luteolin insertion accounted for 75%, 85%, and 75% of all cases for the confirmations of luteolin-β-CD, luteolin-γ-CD, and luteolin-β-CD-MOF, respectively. The chromone ring of luteolin insertion accounted for most of the cases for luteolin-γ-CD-MOF. Luteolin can be fully inserted into β-CD-MOF and γ-CD-MOF, but can only be partially inserted into β-CD and γ-CD. The conformations obtained in the molecular dynamics analysis were summarized in Table 2.

**Table 2:** The conformations of the four complexes, as determined by molecular dynamics analysis.

	Chromone ring insertion	Phenyl ring insertion	Total insertion
Luteolin-β-CD	25%	75%	
Luteolin-γ-CD	15%	85%	
Luteolin-β-CD-MOF	25%	75%	✓
Luteolin-γ-CD-MOF	62%	38%	✓

The binding free energy of luteolin-β-CD-MOF was the lowest (−13.6070 kcal/mol), indicating the most stable structure. Luteolin-γ-CD-MOF had the second most stable structure, with a free binding energy of −13.0973 kcal/mol. The binding free energies of luteolin-γ-CD and luteolin-β-CD were − 11.9834 kcal/mol and − 11.2048 kcal/mol, respectively (Table 3).

**Table 3:** The molecular docking results of 4 complexes.

	Luteolin-β-CD (kcal/mol)	Luteolin-γ-CD (kcal/mol)	Luteolin-β-CD-MOF (kcal/mol)	Luteolin-γ-CD-MOF (kcal/mol)
Binding free energy	−11.2048	−11.9834	−13.6070	−13.0973
Binding free energy subitem				
Electric charge force	−3.2787	−9.5497	−7.9331	−5.9533
Van der Waals force	−11.7634	−7.8423	−11.5225	−13.0344
Interference of solvent	3.8408	5.4051	5.8451	5.8904

The drug-loading process involves multiple forces. The van der Waals force had a greater effect than the electric charge force (hydrogen bond and coordination bond) on maintaining the host-guest system for luteolin-β-CD. In contrast, the electric charge force had a greater effect than the van der Waals force on luteolin-γ-CD. The effect of the solvent on luteolin-γ-CD was more significant than that of luteolin-β-CD, which occurred because the γ-CD molecule was larger than the β-CD molecule. The electric charge force of luteolin-γ-CD was greater than that of luteolin-β-CD, which indicated that more hydrogen bonds were formed in luteolin-γ-CD. These findings suggested that luteolin-γ-CD was more stable than luteolin-β-CD.

Among all the forces tested, the van der Waals forces had the strongest effect on luteolin-β-CD-MOF, followed by the electric charge force. The binding free energy of luteolin-γ-CD-MOF was −13.0973 kcal/mol, which was attributed to the van der Waals force was −13.0344 kcal/mol and the electric charge force was −5.9533 kcal/mol. Because of steric hindrance, the effect of the solvent on luteolin-γ-CD-MOF was more prominent than that on the other materials. Additionally, the van der Waals force was stronger in luteolin-γ-CD-MOF than in luteolin-β-CD-MOF, while the electric charge force showed the opposite patterns. These results indicated that the driving forces for luteolin-β-CD-MOF and luteolin-γ-CD-MOF were different.

The binding free energies of luteolin-CD-MOFs were lower than those of luteolin-CDs, which indicated that luteolin-CD-MOFs were more stable than luteolin-CDs. β-CD-MOF showed a greater affinity for luteolin than γ-CD-MOF, which suggested that β-CD-MOF had more suitable properties for hosting luteolin. Several reasons contributed to these results. Firstly, as β-CD-MOF has a complicated microstructure and is stretchable, the layered architecture can be unfolded to bind drugs under the thermal motion of solvent and drug molecules (Hu et al., 2022). Drug clusters can also be formed in the cavities of β-CD-MOF. Secondly, the surface of β-CD-MOF is rough, as shown in the SEM images, suggesting that its surface can also interact with drugs. Moreover, the presence of luteolin in the β-CD cavity was the optimal conformation for luteolin-β-CD-MOF, which indicated that the structure of β-CD-MOF changed partially during the loading process. This structural alteration was influenced by the movement of the solvent and drug molecules. These results were similar to those obtained by PXRD and TGA in this study. These factors contributed to the effective loading of luteolin into β-CD-MOF.

In conclusion, the driving forces for the formation of the four complexes differed, and β-CD-MOF was found to be the most suitable carrier for luteolin. The van der Waals force and the electric charge force played key roles in balancing the luteolin-β-CD-MOF system.

### 3.7 Antioxidant activity

**Fig. 5:**
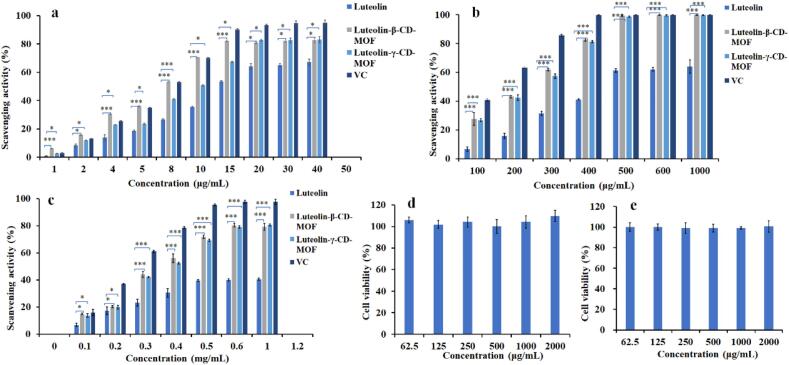
DPPH radical scavenging activities (a), ABTS cationic radical scavenging activities (b) and O_2_^-•^ scavenging activities (c) of luteolin, VC, luteolin-β-CD-MOF, and luteolin-γ-CD-MOF. Cell viabilities of β-CD-MOF (d) and γ-CD-MOF (e) on L02 cells (Data was presented as mean ± SD, *n* = 6, * represents a significant difference compared with the pure luteolin, *p < 0.05, **p < 0.01, ***p < 0.001).

DPPH is commonly used as a free radical to evaluate the reduction of substances. We tested the DPPH radical scavenging activities of luteolin, VC, luteolin-β-CD-MOF, and luteolin-γ-CD-MOF. As one can see from Fig. 5a, when the concentration of luteolin was <20 μg/mL, the radical scavenging activity of raw luteolin was dose-dependent. And the highest radical scavenging activity of luteolin was about 64%. However, both the radical scavenging activity of luteolin-β-CD-MOF and luteolin-γ-CD-MOF was 82% at 20 μg/mL. Luteolin-β-CD-MOF and luteolin-γ-CD-MOF had higher DPPH scavenging activities.

The ABTS cationic radical scavenging activities of luteolin, VC, luteolin-β-CD-MOF, and luteolin-γ-CD-MOF were shown in Fig. 5b. When the concentration of luteolin lower than 500 μg/mL, it also showed dose-dependent scavenging activity. The highest scavenging activity of raw luteolin was 61% at concentrations of 500 μg/mL, but both that of luteolin-β-CD-MOF and luteolin-γ-CD-MOF was 100% at a concentration of 500 μg/mL. These results indicated that luteolin-β-CD-MOF and luteolin-γ-CD-MOF can significantly improve the ABTS cationic scavenging activity.

O_2_^-•^ is an active oxygen species with a strong oxidation ability. The overproduction of O_2_^-•^ can damage cells. We determined the O_2_^-•^ scavenging activities of luteolin, VC, luteolin-β-CD-MOF, and luteolin-γ-CD-MOF (Fig. 5c). The O_2_^–•^ scavenging ability increased with increasing concentrations of luteolin. The highest O_2_^-•^ scavenging activity of 80% was recorded at a concentration of 0.6 mg/mL for luteolin-β-CD-MOF and luteolin-γ-CD-MOF. In contrast, the scavenging activity of raw luteolin was only 40%. This finding indicated that luteolin-β-CD-MOF and luteolin-γ-CD-MOF can improve O_2_^-•^ scavenging activity of luteolin.

Free radicals can act as pathogenic factors and are closely related to various diseases. Luteolin is a flavonoid commonly found in herbs. The antioxidant activity of luteolin contributes to its pharmacological effects (Wang et al., 2019). Therefore, the antioxidant activity of luteolin needs to be investigated. Our results showed that luteolin-β-CD-MOF and luteolin-γ-CD-MOF increased the DPPH, ABTS, and O_2_^-•^ scavenging activities of luteolin. The unique microstructures of CD-MOFs contributed to this phenomenon, they prevented the entry of oxidant molecules due to the steric hindrance (Xiong et al., 2019). Furthermore, an increase in the dissolution of luteolin-β-CD-MOF and luteolin-γ-CD-MOF also increased the antioxidant activity (Jia et al., 2024).

### 3.8 Cytotoxicity assay

The biocompatibility of β-CD-MOF and γ-CD-MOF plays a key role in their applications. Thus, we analyzed their safety profiles in human hepatocytes (L02 cells). The difference in cells growth exposed to different concentrations of β-CD-MOF or γ-CD-MOF was not significant (Fig. 5d-e). As adding β-CD-MOF or γ-CD-MOF to cells did not inhibit cells growth, we considered β-CD-MOF and γ-CD-MOF to be safe for L02 cells. Based on these findings, we inferred that β-CD-MOF and γ-CD-MOF can be safely used in the relevant medical studies.

### 3.9 *In vivo* pharmacokinetic studies

#### 3.9.1 Pharmacokinetics study of pure luteolin (Y) and luteolin-β-CD-MOF (Z) in healthy rats (C)

**Fig. 6:**
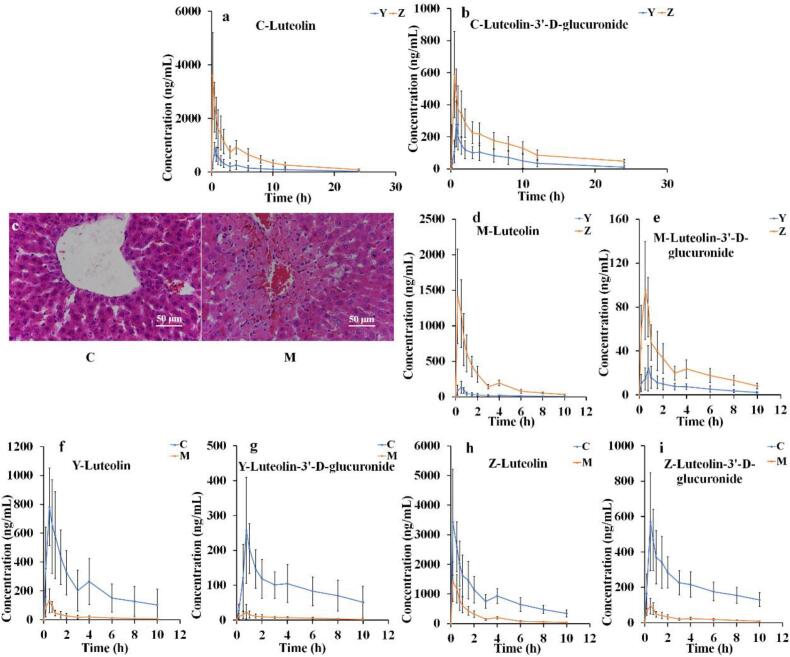
The plasma concentration-time curves (a-b) of luteolin and luteolin-3’-D-glucuronide in healthy rats (C) after oral administration of pure luteolin (Y) and luteolin-β-CD-MOF (Z) (Data was presented as mean ± SD, n = 6). Histopathological changes (c) in livers of C (healthy rats) and M (liver injured rats) stained with HE (200×). The plasma concentration-time curves (d-e) of luteolin in liver injured rats (M) after orally administration pure luteolin (Y) and luteolin-β-CD-MOF (Z) (Data was presented as mean ± SD, n = 6). The plasma concentration-time curves (f-i) of luteolin and luteolin-3’-D-glucuronide in heathy rats (C) and liver injured rats (M) after orally administration pure luteolin (Y) and luteolin-β-CD-MOF (Z) (Data was presented as mean ± SD, n = 6).

The synthesis cost of β-CD-MOF was lower than that of γ-CD-MOF. And as determined by the molecular simulation, β-CD-MOF was the most suitable carrier for luteolin. Therefore, luteolin-β-CD-MOF was selected for the subsequent *in vivo* studies. Luteolin-3’-D-glucuronide is the predominant metabolite of luteolin *in vivo* (Wu et al., 2015), and its level reflects the absorption of luteolin. Therefore, we codetermined the levels of luteolin and luteolin-3’-D-glucuronide in rats after oral administration of luteolin-β-CD-MOF and raw luteolin, respectively. The mean plasma concentration-time curves of luteolin and luteolin-3’-D-glucuronide were shown in Fig. 6a and Fig. 6b. The pharmacokinetic parameters were summarized in Table 4. The plasma concentration and AUC _0–24 h_ of luteolin and luteolin-3’-D-glucuronide from Z were greater than those recorded in the raw form, suggesting that luteolin-β-CD-MOF improved luteolin's oral absorption. The bioavailability of luteolin from luteolin-β-CD-MOF was 4.04-fold greater than that from crude luteolin. The bioavailability of luteolin-3’-D-glucuronide from luteolin-β-CD-MOF was 2.55-fold greater than that from crude luteolin. These results indicated that β-CD-MOF is an effective carrier that can improve the bioavailability of luteolin in healthy rats.

**Table 4:** The pharmacokinetic parameters of pure luteolin (Y) and luteolin-β-CD-MOF (Z) in heathy rats (C) (Data was presented as mean ± SD, *n* = 6, *p < 0.05 *versus* Y as reference).

	C _max_ (ng/mL)	AUC _0__–__24 h_ (ng/mL*h)	Relative bioavailability (%)
C-Y-luteolin (reference)	855.27 ± 253.07	3035.79 ± 1232.16	404.10 ± 78.62
C-*Z*-luteolin	3596.44 ± 1618.03*	11,729.30 ± 3499.59*	
C-Y-luteolin-3’-D-glucuronide (reference)	277.89 ± 141.23	1328.13 ± 613.35	255.42 ± 67.61
C-*Z*-luteolin-3’-D-glucuronide	588.74 ± 268.69*	3201.90 ± 902.20*	

#### 3.9.2 Pharmacokinetics study of pure luteolin (Y) and luteolin-β-CD-MOF (Z) in rats with liver injury (M) induced by acetaminophen

Luteolin can prevent liver injury (Tai and Zhang, 2015), but its pharmacokinetic behavior in rats with liver injury has not been studied. Thus, we investigated the pharmacokinetic behavior of luteolin in rats with liver injury. To understand the histopathological changes in liver, liver tissues were collected after 1000 mg/kg acetaminophen was administered i.p. at 24 h, and stained with HE. We found that healthy rats had a normal liver cell structure, the morphology of the hepatic sinus was clear. And inflammatory responses were absent (Fig. 6c). The pyknosis and necrosis of liver cell nuclei were observed. Inflammatory cells infiltrated the livers of the rats that were administered 1000 mg/kg acetaminophen i.p., which indicated that the rats suffered liver injury.

The mean plasma concentration-time profiles of Y and Z in rats with liver injury were shown in Fig. 6d and Fig. 6e, and the pharmacokinetic parameters were summarized in Table 5. For the Z group, C _max_ increased from 159.52 to 1465.49 ng/mL and the AUC _0–10 h_ increased from 228.41 to 2300.66 ng/mL*h for luteolin in rats with liver injury compared to the Y group. The bioavailability of luteolin from luteolin-β-CD-MOF was 11.07-fold greater than that of crude luteolin in liver injury rats. For the Z group, C _max_ increased from 25.03 to 102.82 ng/mL and the AUC _0–10 h_ increased from 71.34 to 250.71 ng/mL*h for luteolin-3’-D-glucuronide in rats with liver injury compared to the Y group. The bioavailability of luteolin-3’-D-glucuronide from luteolin-β-CD-MOF was 3.95-fold greater than that from crude luteolin in rats with liver injury. These results suggested that luteolin-β-CD-MOF improved the oral absorption of luteolin in liver injured rats.

**Table 5:** The pharmacokinetic parameters of pure luteolin (Y) and luteolin-β-CD-MOF (Z) in liver injured rats (M) (Data was presented as mean ± SD, n = 6, *p < 0.05 *versus* Y as reference).

	C _max_ (ng/mL)	AUC _0__–__10 h_ (ng/mL*h)	Relative bioavailability (%)
M-Y-luteolin (reference)	159.52 ± 62.78	228.41 ± 82.84	1107.44 ± 514.54
M-Z-luteolin	1465.49 ± 617.48*	2300.66 ± 680.89*	
M-Y-luteolin-3’-D-glucuronide (reference)	25.03 ± 2.16	71.34 ± 35.48	394.95 ± 147.41
M-Z-luteolin-3’-D-glucuronide	102.82 ± 43.53*	250.71 ± 65.29*	

Luteolin-β-CD-MOF increased the oral absorption of luteolin in healthy and liver injured rats for several reasons. Firstly, the higher dissolution rate of luteolin-β-CD-MOF saturated the metabolic enzymes and increased the content of drugs entering the blood circulation (Gilley et al., 2017). Secondly, the increased dissolution of luteolin-β-CD-MOF promoted the absorption of luteolin molecules in the gastrointestinal tract (Moretton et al., 2014). Moreover, the cations in CD-MOF can also improve the pharmacokinetics (Hamedi et al., 2021a, Hamedi et al., 2021b). Thus, β-CD-MOF can be used to increase the bioavailability of luteolin.

The pharmacokinetic parameters of luteolin and luteolin-3’-D-glucuronide were significantly different between healthy and liver injured rats. The changes in the pharmacokinetic behavior were presented in Fig. 6f-i and the pharmacokinetic parameters were summarized in Table 6. Y in healthy rats was used as a reference, the relative bioavailability of luteolin from Y in rats with liver injury was reduced to 10.73%, and the relative bioavailability of luteolin-3’-D-glucuronide from Y in rats with liver injury was reduced to 8.25%. At the same time, Z in healthy rats was used as a reference, the relative bioavailability of luteolin from Z in rats with liver injury was reduced to 25.69%, while that of luteolin-3’-D-glucuronide from Z in rats with liver injury was reduced to 11.82%. These results indicated that the absorption of luteolin was impaired after a high dose of acetaminophen was administered, which induced the dysfunction in hepatocytes, leading to a decrease in the relative bioavailability of luteolin.

**Table 6:** The pharmacokinetic parameters of pure luteolin (Y) and luteolin-β-CD-MOF (Z) in heathy rats (C) and liver injured rats (M) (Data was presented as mean ± SD, *n* = 6, *p < 0.05 *versus* Y as reference).

	C _max_ (ng/mL)	AUC _0__–__10 h_ (ng/mL*h)	Relative bioavailability %
Y-C-luteolin (reference)	855.27 ± 253.07	2415.17 ± 1245.69	10.73 ± 3.90
Y-M-luteolin	159.52 ± 62.78*	228.41 ± 82.84*	
*Z*-C-luteolin (reference)	3596.44 ± 1618.03	9034.23 ± 2772.79	25.69 ± 3.71
Z-M-luteolin	1465.49 ± 617.48*	2300.66 ± 680.89*	
Y-C-luteolin-3’-D-glucuronide (reference)	277.89 ± 141.23	960.95 ± 439.21	8.25 ± 3.85
Y-M-luteolin-3’-D-glucuronide	25.03 ± 20.16*	71.34 ± 35.48*	
Z-C-luteolin-3’-D-glucuronide (reference)	588.74 ± 268.69	2187.36 ± 678.47	11.82 ± 2.08
Z-M-luteolin-3’-D-glucuronide	102.82 ± 43.53*	250.71 ± 65.29*	

### 3.10 *In vivo* liver protective effect of luteolin (Y) and luteolin-β-CD-MOF (Z) in rats with liver injury induced by acetaminophen

#### 3.10.1 Liver histological analysis

**Fig. 7:**
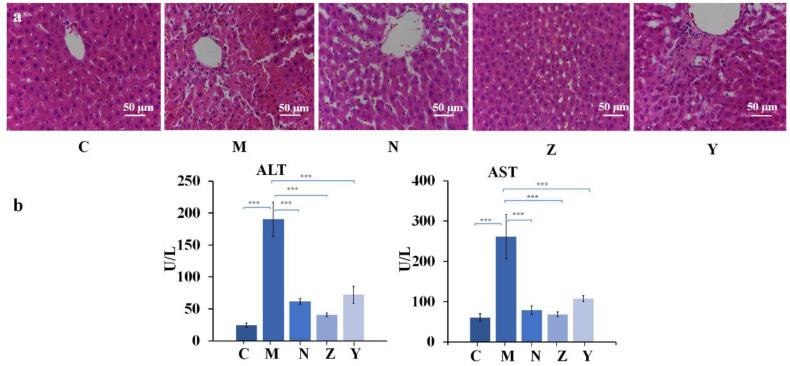
Histopathological changes in rat livers of C, M, N, Z and Y groups stained with HE (200×) (a). The biochemical analysis results in rats of C, M, N, Z and Y groups (b) (Data was presented as mean ± SD, n = 6, * represents a significant difference compared with the M group, *p < 0.05, **p < 0.01, ***p < 0.001).

The morphological changes in each group were shown in Fig. 7a. The cell structure of the rats in C group was normal. However, pyknosis and necrosis of liver nuclei, and many inflammatory infiltrations were observed in M group, which indicated that acetaminophen caused liver damage in rats. Pretreatment with luteolin or *N*-acetylcysteine alleviated liver injury, indicated luteolin and *N*-acetylcysteine can protect the liver. Furthermore, luteolin-β-CD-MOF exhibited a better liver protection effect than luteolin.

#### 3.10.2 Biochemical analysis

The serum ALT and AST activities were significantly increased in M group than in C group (Fig. 7b), which indicated that the acetaminophen-induced liver injury rat model was established. The serum ALT and AST activities were lower in N, Z, and Y groups than their corresponding activities in M group, which suggested that luteolin and *N*-acetylcysteine can protect the liver. Additionally, pre-administration of luteolin-β-CD-MOF had a greater hepatoprotective effect than luteolin. This occurred for several reasons. Firstly, luteolin has antioxidant activity and can eliminate free radicals and restore the redox homeostasis (Kuo et al., 2011). Based on these effects, luteolin can inhibit the pathological changes during liver injury. The improvement of antioxidant activity can promote its anti-liver injury effect. Secondly, luteolin-β-CD-MOF increased the bioavailability of luteolin, which implied that β-CD-MOF strategy facilitated the absorption of more luteolin molecules into the bloodstream. Consequently, it showed a better pharmacological performance. And the molecular mechanism of liver protective effect of luteolin-β-CD-MOF has been elucidated in other study (Yang et al., 2024).

As shown in Table 7, several strategies have been adopted to improve the properties of luteolin. In comparison to other reported orally solubilization techniques for luteolin in literature, luteolin-β-CD-MOF has many advantages. Firstly, the preparation method for luteolin-β-CD-MOF is simple and eco-friendly, requiring minimal cost and no specialized equipment, unlike nanomaterials or liposomes. Moreover, β-CD-MOF is a safe and non-toxic edible material. Most significantly, luteolin-β-CD-MOF has been shown to increase the bioavailability of luteolin by approximately 4.04 and 11.07 times in healthy rats and liver injured rats compared to raw luteolin, and has good anti-liver injury effect. To summarize, the results of pharmacokinetic and pharmacodynamic studies in rats showed that β-CD-MOF is a suitable drug carrier for oral drug delivery.

**Table 7:** Comparison of other solubilizing studies about luteolin on various materials.

NO.	Carrier	Dosage Form	Highlights	Method	Reference
1	Isoniazid	Cocrystal	The bioavailability of luteolin was enhanced by 2.70 times.		Luo and Chen, 2019
2	Caffeine	Cocrystal	The bioavailability of luteolin was enhanced by 1.40 times.		Luo and Chen, 2019
3	D-α-tocopherol polyethylene glycol 1000 succinate	Microemulsion	The antioxidant activity of luteolin was enhanced.		Zheng et al., 2023
4	Zein-Caseinate	Nanoparticle	The bioavailability of luteolin was enhanced by 2.92 times.	Shape memory films method (under reduced pressure to remove the tetrahydrofuran)	Xu et al., 2023
5	Whey protein isolate	Liposome	The thermal stability and antioxidant activity of luteolin were improved.	Thin-film evaporation method	Huang et al., 2024
6	Zein-gum arabic-tea polyphenols	Nanoparticle	The antioxidant activity and sustainable release capacity of luteolin were improved.	Anti-solvent precipitation method	Li et al., 2023
7	Zein	Microparticle	The antioxidant activity and dissolution rate of luteolin were improved.	Supercritical fluids assisted technique	Palazzo et al., 2019
8	Vitamin E d-α-tocopherol acid polyethylene glycol 1000 succinate	Liposome	The cellular uptake, the cytotoxicity in A549 cells, tumor inhibition of luteolin were improved.	Film-dispersion method	Li and Cheng, 2016
9	Caprylic/capric triglyceride, polyoxyl 35 hydrogenated castor oil and polyethylene glycol 400	Supersaturatable self-nanoemulsifying drug delivery system	The bioavailability of luteolin was enhanced by 2.20 times compared to that of the conventional self-nanoemulsifying drug delivery system.	Self-micro-emulsifying technique	Zhang et al., 2020
10	Hybrid polylactic acid/Eudragit L100	Nanoparticle	Compared to luteolin suspension, the bioavailability of luteolin was enhanced by 2.61 times.	Anti-solvent precipitation and vacuum freeze-dry technology	Elmowafy et al., 2021
11	Polylactic acid/polyethylene glycol	Phospholipid complex	The bioavailability of luteolin was enhanced by 3.54 times.		Zhou et al., 2024
12	Chitosan	Phyto-cubosome	The sustained drug release profile, anti-oxidant activity, and transcorneal permeation of luteolin were improved.	Hydrotrope technique	Omran et al., 2024
13	Choline oleate	Nano-dispersion	The solubility, antioxidant activity, and antibacterial activity of luteolin were improved.	Emulsification and lyophilization processes	Shimul et al., 2022
14	Plant exosome- derived from sesame leaves	Nanoparticle	The solubility, antioxidant ability and bioavailability of luteolin were improved.	Liquid anti-solvent precipitation and vacuum freeze-dry technology	Jiang et al., 2024
15	Soybean protein	Liposome	The solubility, thermal stability, DPPH scavenging activity of luteolin were improved.	Ultrasonic pretreatment	Sun et al., 2022
16	β-cyclodextrin and pluronic F127	Host-guest complex	The solubility, dissolution rate, and antioxidant activity of luteolin were improved.	Microwave irradiation technique	Alshehri et al., 2020

## 4 Conclusion

In this study, we found that β-CD-MOF is non-toxic and can be used as a carrier to load luteolin. We developed a simple, eco-friendly, and effective method to improve the dissolution, antioxidant activity, bioavailability, and hepatoprotective effect of luteolin. The results of several characterization tests showed that luteolin was successfully loaded into β-CD-MOF. Moreover, hydrogen bonds and van der Waals forces were the main driving forces for luteolin-β-CD-MOF, as determined by molecular docking techniques. Among the four complexes, luteolin-β-CD-MOF had the lowest binding free energy and the highest loading capacity. These results suggested that β-CD-MOF was the most suitable carrier for luteolin. The results of *in vitro* tests showed that luteolin-β-CD-MOF increased the dissolution and antioxidant activity of luteolin. The results of *in vivo* tests showed that luteolin-β-CD-MOF improved the bioavailability of luteolin in healthy rats and rats with acetaminophen-induced liver injury. Moreover, luteolin-β-CD-MOF showed better hepatoprotective effects than raw luteolin in rats with acetaminophen-induced liver injury. This study elucidated the mechanism of β-CD-MOF loading luteolin, explored more applications for β-CD-MOF, and provided new strategies to prevent acetaminophen-induced liver injury.

## CRediT authorship contribution statement

**Dan Yang:** Writing – original draft, Conceptualization. **Min Zhao:** Methodology. **Yihe Huang:** Software. **Liwen Chen:** Supervision. **Jiqin Fang:** Validation. **Jiaonan Liu:** Visualization. **Miao Wang:** Data curation. **Chunjie Zhao:** Resources.

## Declaration of competing interest

The authors declare that they have no competing financial interests or personal relationships that could have appeared to influence the work reported in this paper.

The authors declared no potential conflict of interest with respect to the research, authorship, and/or publication of this article.

## Data Availability

Data will be made available on request.

## Appendix A Supplementary data

Supplementary Materials Image 1
