# Supplementary material for: β-Cyclodextrin metal-organic framework as a green carrier to improve the dissolution, bioavailability, and liver protective effect of luteolin
Source: Int J Pharm X. 2024 Apr 26;7:100250. doi: 10.1016/j.ijpx.2024.100250 (PMC11070924; doi:10.1016/j.ijpx.2024.100250)
Supplement: Supplementary file 1 — Supplementary Materials [file mmc1.docx]

Supplementary Material

1. **Supplementary Data**
   1. **. Optimizing of preparation conditions for luteolin-β-CD-MOF**

The impacts of varying reaction temperatures, mass ratios, and reaction time on the preparation for luteolin-β-CD-MOF were investigated by determining the accumulative dissolution amount in 900 mL of distilled water. Firstly, to study the effect of reaction temperature on the preparation for luteolin-β-CD-MOF, the prepared temperature of 30 ℃, 40 ℃, and 50 ℃ were investigated at the mass ratio of 1:7 (luteolin: β-CD-MOF, w/w) reacted for 9 h in ethanol, respectively. Subsequently, the impact of varying reaction time (2, 6, 8, 10, 12, and 48 h) on the preparation for luteolin-β-CD-MOF was investigated at a mass ratio of 1:7 (luteolin: β-CD-MOF, w/w) in ethanol at 40 ℃. Finally, the influence of different mass ratios (1:5, 1:6, 1:7, 1:10, 1:15, luteolin: β-CD-MOF, w/w) on the preparation for luteolin-β-CD-MOF was studied under gentle stirring for 10 h at 40 ℃ in ethanol. The optimal temperature, mass ratio and reaction time of the preparation for luteolin-β-CD-MOF were determined based on the results obtained from dissolution tests.

- 1. **Optimizing of preparation conditions for** **luteolin-γ-CD-MOF**

The impacts of varying reaction temperatures, mass ratios, and reaction time on the preparation for luteolin-γ-CD-MOF were also investigated by determining the accumulative dissolution amount in 900 mL of distilled water. Firstly, the impact of reaction temperature (30 ℃, 40 ℃, and 50 ℃) on the preparation for luteolin-γ-CD-MOF was investigated with a mass ratio of 1:4 (luteolin: γ-CD-MOF, w/w) for 36 h in ethanol. Secondly, to examine the impact of mass ratio on the preparation for luteolin-γ-CD-MOF, various ratios (1:1, 1:4, 1:5, 1:6, 1:7, 1:8, 1:10, 1:12, luteolin: γ-CD-MOF, w/w) were studied with gentle stirring for 30 h at 40 ℃ in ethanol. Finally, to study the effect of reaction time on the preparation for luteolin-γ-CD-MOF, the reaction time of 2, 9, 12, 48, 72, 84 h were investigated with the mass ratio of 1:8 (luteolin: γ-CD-MOF, w/w) at 40 ℃ in ethanol, respectively. The optimal temperature, mass ratio and reaction time of the preparation for luteolin-γ-CD-MOF were determined based on the results obtained from dissolution tests.

1. **Supplementary Table**

Table S1 The procedures of DPPH, ABTS, and O_2_^-•^ scavenging activities experiments.

|  | DPPH scavenging activity | | | | ABTS scavenging activity | | | | O_2_^-•^ scavenging activity | | | |
| --- | --- | --- | --- | --- | --- | --- | --- | --- | --- | --- | --- | --- |
|  | Sample solution (mL) | DPPH solution (mL) | Water  (mL) | Ethanol  (mL) | Sample solution (mL) | ABTS solution (mL) | Water (mL) | Methanol  (mL) | Sample solution (mL) | Tris-HCL solution (mL) | Pyrogallol  solution (mL) | Water  (mL) |
| A *_sample_* | 2 | 2 |  |  | 0.1 | 4 |  |  | 1 | 4 | 0.2 |  |
| A *_blank_* |  | 2 | 2 |  |  | 4 | 0.1 |  |  | 4 | 0.2 | 1 |
| A *_control_* | 2 |  |  | 2 | 0.1 |  |  | 4 | 1 | 4 |  | 0.2 |

1. **Supplementary Figure**

**
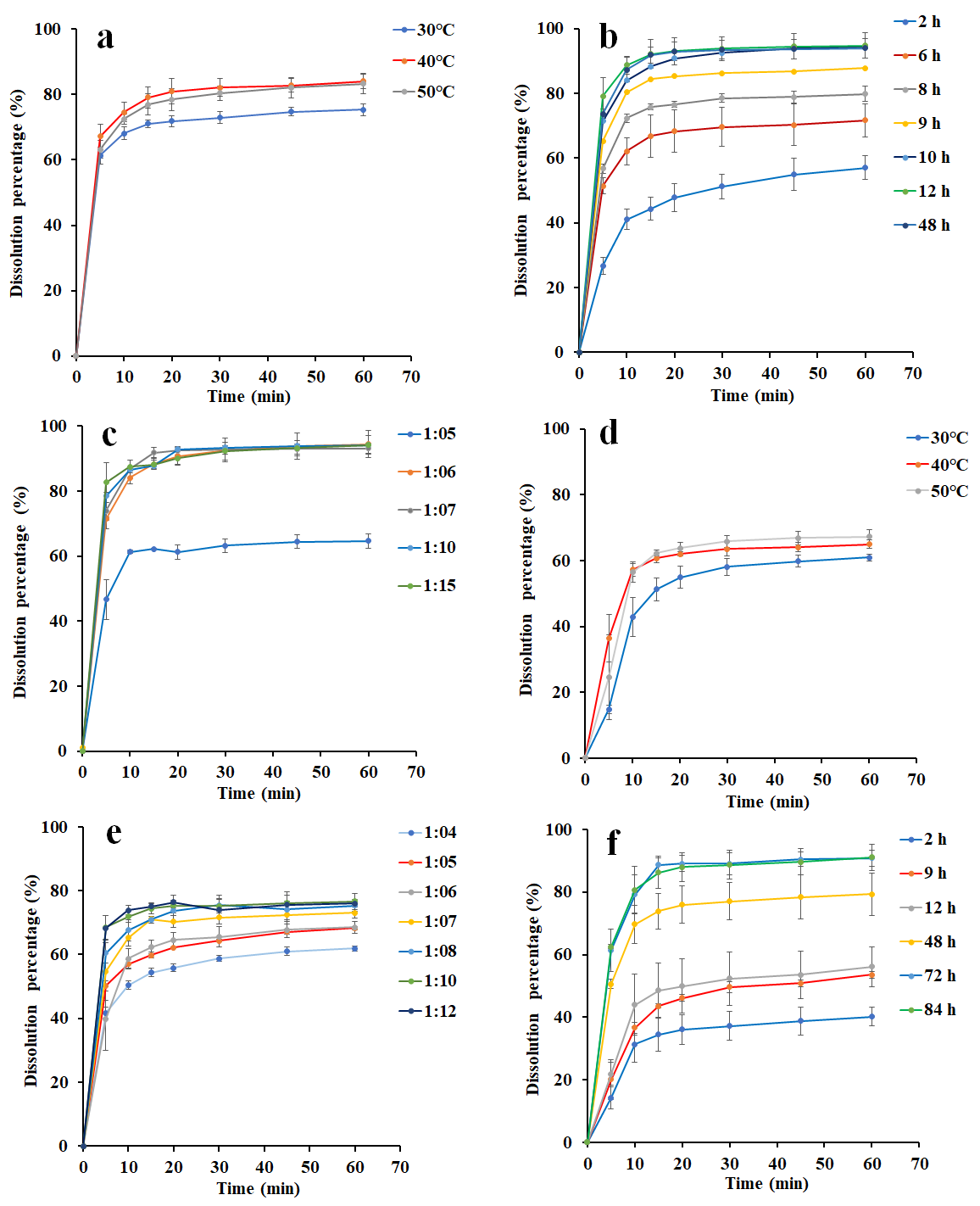
**

Fig. S1. The dissolution profiles at different temperatures (a), different reaction time (b) and different mass ratios (luteolin: β-CD-MOF, w/w) (c) for luteolin-β-CD-MOF in distilled water. The dissolution profiles at different temperatures (d), different mass ratios (luteolin: **γ**-CD-MOF, w/w) (e), and different reaction time (f) for luteolin-γ-CD-MOF in distilled water (Data was presented as mean± SD).
